# The SHED-IT community trial study protocol: a randomised controlled trial of weight loss programs for overweight and obese men

**DOI:** 10.1186/1471-2458-10-701

**Published:** 2010-11-16

**Authors:** Philip J Morgan, Clare E Collins, Ronald C Plotnikoff, Patrick McElduff, Tracy Burrows, Janet M Warren, Myles D Young, Nina Berry, Kristen L Saunders, Elroy J Aguiar, Robin Callister

**Affiliations:** 1 School of Education, University of Newcastle, Callaghan Campus, Australia; 2 School of Health Sciences, University of Newcastle, Callaghan Campus, Australia; 3 School of Medicine and Public Health, University of Newcastle, Callaghan Campus, Australia; 4 Danone Baby Nutrition, White Horse Park Business Park, Trowbridge, Wiltshire, BA14 OXQ, UK; 5 School of Biomedical Sciences, University of Newcastle, Callaghan Campus, Australia

## Abstract

**Background:**

Obesity is a major cause of preventable death in Australia with prevalence increasing at an alarming rate. Of particular concern is that approximately 68% of men are overweight/obese, yet are notoriously difficult to engage in weight loss programs, despite being more susceptible than women to adverse weight-related outcomes. There is a need to develop and evaluate obesity treatment programs that target and appeal to men. The primary aim of this study is to evaluate the efficacy of two relatively low intensity weight loss programs developed specifically for men.

**Methods and Design:**

The study design is an assessor blinded, parallel-group randomised controlled trial that recruited 159 overweight and obese men in Newcastle, Australia. Inclusion criteria included: BMI 25-40 (kg/m^2^); no participation in other weight loss programs during the study; pass a health-screening questionnaire and pre-exercise risk assessment; available for assessment sessions; access to a computer with e-mail and Internet facilities; and own a mobile phone. Men were recruited to the SHED-IT (Self-Help, Exercise and Diet using Internet Technology) study via the media and emails sent to male dominated workplaces. Men were stratified by BMI category (overweight, obese class I, obese class II) and randomised to one of three groups: (1) SHED-IT *Resources *- provision of materials (DVD, handbooks, pedometer, tape measure) with embedded behaviour change strategies to support weight loss; (2) SHED-IT *Online *- same materials as SHED-IT *Resources *plus access to and instruction on how to use the study website; (3) Wait-list Control. The intervention programs are three months long with outcome measures taken by assessors blinded to group allocation at baseline, and 3- and 6-months post baseline. Outcome measures include: weight (primary outcome), % body fat, waist circumference, blood pressure, resting heart rate, objectively measured physical activity, self-reported dietary intake, sedentary behaviour, physical activity and dietary cognitions, sleepiness, quality of life, and perceived sexual health. Generalised linear mixed models will be used to assess all outcomes for the impact of group (*Resources*, *Online*, and *Control*), time (treated as categorical with levels baseline, 3-months and 6-months) and the group-by-time interaction. These three terms will form the base model. 'Intention-to-treat' analysis will include all randomised participants.

**Discussion:**

Our study will compare evidence-based and theoretically driven, low cost and easily disseminated strategies specifically targeting weight loss in men. The SHED-IT community trial will provide evidence to inform development and dissemination of sustainable strategies to reduce obesity in men.

**Trial Registration:**

Australian New Zealand Clinical Trials Registry (ACTRN12610000699066)

## Background

Obesity is a major cause of preventable death and is associated with a range of negative physiological and psychological consequences [1]. In addition, obesity-related health care costs are substantial [2]. Approximately two thirds of Australian men are overweight/obese [3] and men are more likely to be so than women in every age group [4]. Men are at higher risk for developing metabolic syndrome compared to females and obese men are six times more likely to develop metabolic syndrome compared to those of healthy weight [5]. Overweight men have greater abdominal adiposity than women, which further increases health risks including cardiovascular disease (CVD) [6]. Consequently, men may derive greater risk factor reduction from weight loss than women. Obesity in men represents a significant community health problem that requires an urgent and informed response.

A major public health challenge is to design weight loss programs that engage men. Compared to women, men are less likely to perceive themselves as overweight [7], attempt weight loss, or participate in weight loss programs [8]. Although reasons for this lack of engagement are not well established, it appears that men perceive too many barriers and/or currently available programs do not appeal to them [9]. Few studies have been conducted in men. In Australia, the *Gut-Buster *study was unique when published 14 years ago [10] but was reported to be expensive to run and had limited sustainability [11]. It has been documented that men desire weight loss programs that are convenient, provide individualised feedback, and include participants with whom they identify [9]. While intensive group programs with weekly visits can be important components of effective treatment, they are not practical for the time-poor and may not appeal to men in particular. Men are generally not enthusiastic about attending structured face-to-face weight loss programs [9,12]. Consequently, alternative treatment approaches such as the Internet and/or interactive resources may be more appealing and afford greater accessibility, anonymity and convenience [13].

Over the past 10-15 years, Internet-based interventions for weight management have been designed and evaluated [13-15]. The Internet is accessible 24 hours a day, allowing convenient usage and compatibility with busy schedules [14]. However, the use of the Internet for obesity treatment is under-explored, especially in Australia, relative to the rapid uptake of the Internet in the home environment. From 1998 to 2008, home access to the Internet more than quadrupled from 16% to 67% [16]. Notably, in Australia, men are more likely to use the Internet than women [17]. The Internet has considerable potential to deliver weight management programs and provide an alternative treatment that minimises the participant burden associated with group sessions and clinic visits [18].

Recent systematic reviews of online weight loss randomised controlled trials (RCTs) have concluded that weight loss programs can be effectively delivered over the Internet [19-21]. However, limitations of previous studies include no 'intention-to-treat' analysis, no assessor blinding, follow-up measures based only on participants' self-report, moderate retention rates, and insufficient follow-up beyond immediate post intervention assessments. In addition, these reviews have recommended high quality studies need to be carried out in specific sub-groups of the population [19,20]. For example, the generalisability of the findings of most online studies has been questioned as they have recruited predominantly women [19]. Few weight loss studies have been conducted in men [10,22] and to the authors knowledge, the pilot study of the SHED-IT (Self-Help, Exercise and Diet using Internet Technology) program is the only study to have evaluated an online, weight loss RCT in men.

### The SHED-IT pilot study

In 2007/2008, we conducted the first randomised controlled trial of an online weight loss program that targeted men exclusively [23-26]. Sixty-five overweight/obese male staff and students of the University of Newcastle were recruited and randomly assigned to either an (i) Internet group or (ii) Information only group. Both groups received one face-to-face information session and a program booklet. Internet group participants were instructed to use the study website for three months. 'Intention-to-treat' analysis revealed significant and sustained weight loss of -5.3 kg at 12 months for the Internet group and -3.1 kg for the Information only group with no significant group difference [26].

Both the Internet and Information only programs were effective, but those participants who complied with the recommended Internet features were able to maintain significantly greater weight loss than those who did not comply [24,26]. A key feature of both programs was the low level of interaction between the researchers and participants, which highlighted the potential of SHED-IT as a cost effective approach to weight loss for men at a population level. The Information only group was an effective low dose treatment option, but we were unable to establish if it was the face-to-face information session, or the provided resources that were most effective.

In summary, our pilot study confirmed the feasibility and preliminary efficacy of the SHED-IT program [24] and addressed many of the limitations identified in previous online studies [15]. We found that low-dose approaches to weight loss can achieve clinically important weight loss in men after one year follow-up [26]. Qualitative analysis by questionnaire and interview highlighted that the men found the program acceptable and in general were satisfied with the program [26]. The process evaluation was congruent with the positive outcomes of the study but identified a number of areas for improvement including strategies to improve online compliance and understanding of website features, and alignment with the theoretical framework. In addition, each of the pilot arms included a face-to-face information session. We now wish to evaluate whether the same effect can be achieved with a DVD, improved support resources and implementation of additional strategies to operationalise key theoretical constructs. Previously, the study was conducted in a convenience sample of academic and non-academic overweight male staff and students of a University and it will now be tested in a larger trial using a community population sample to test generalisability.

### Aims And Hypotheses

Our overall research question is: *Can relatively low dose weight loss programs be effective in achieving weight loss in a community sample of overweight men? *The primary aim is to determine the efficacy of two behavioural weight loss programs designed to be incremental in the extent of interaction and intensity of participation, and differ in mode of delivery in a large community trial. A secondary aim is to compare the effectiveness of the different intervention programs in defined subgroups (e.g., age, education, occupation, marital status, SES).

It is hypothesised that:

(1) Compared to the Wait-list Control, both the SHED-IT *Resources *and *Online *interventions will result in a clinically important and statistically significant (a) reduction in weight and (b) improvement in other important secondary outcomes at 3 and 6 months post-baseline

(2) The SHED-IT *Online *program will result in greater improvements in the primary and secondary outcomes at 3 and 6 months post-baseline compared to the SHED-IT *Resources *program.

## Methods

### Study design

**Figure 1:**
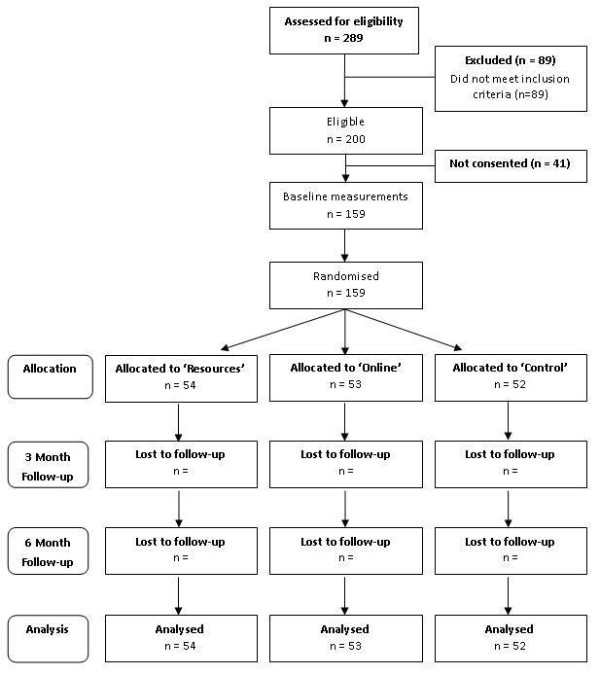
**CONSORT Flowchart describing the progress of participants through the trial**.

The study design is an assessor blinded, parallel-group randomised controlled trial and is summarised in Figure 1. The study has been approved by the University of Newcastle Human Research Ethics Committee and is registered with the Australian New Zealand Clinical Trials Registry (ACTRN12610000699066). Following the initial screening process for inclusion/exclusion (see Table 1), men were stratified by BMI category (overweight, obese class I and obese class II) and randomised to one of three study arms:

**Table 1:** Inclusion and exclusion criteria for the SHED-IT community trial

*Inclusion criteria*	*Exclusion criteria*
* were male;	* had a history of major medical problems such as heart disease or diabetes in the last five years that would prevent them from exercising;
* had a BMI between 25 and 40 (kg/m^2^);	* had orthopaedic or joint problems that would be a barrier to physical activity such as walking;
* agreed to not participate in other weight loss programs during the study;	* had recently lost 5% or more of their body weight;
* passed a health-screening questionnaire and a pre-exercise risk assessment [28];	* were taking medications that are affected by weight loss or had resulted in weight gain or loss in the last 3 months;
* were available for assessment sessions;	* were currently participating in an alternative weight loss program.
* had access to a computer with e-mail and Internet facilities;	
* owned a mobile phone.	

(i) SHED-IT *Resources*

(ii) SHED-IT *Online *- Resources plus Internet

(iii) *Wait-list Control - *6 month wait-list

The design, conduct and reporting of this study will adhere to the Consolidated Standards of Reporting Trials (CONSORT) guidelines [27].

### Participants

Overweight or obese (BMI between 25 and 40 kg/m^2^) men aged 18 to 65 years were recruited in July/August 2010 from the local community of the Hunter Region, New South Wales, Australia. Participants were recruited through advertising (radio, TV, newspapers, University website) using the University media unit and via workplace-based emails and notices. Participants were screened for eligibility via telephone using a standardised protocol. Eligibility criteria are also included in Table 1.

All participants were required to provide informed consent in writing and provide a medical clearance from their general practitioner to participate in the study if they were over 40 years of age [28] or if there were possible health concerns identified in the screening questionnaires.

### Study Interventions

Intensive face-to-face interventions may not be feasible as a health service model for treating obesity and therefore the SHED-IT interventions are being evaluated at two levels of intensity: Resource-based and Online. Our interventions have been informed by: (i) our recent systematic review (meta analyses) [29] examining online weight loss and factors associated with weight loss; (ii) our recent articles on mediators of weight loss in men from the SHED-IT pilot [23] and process evaluation [26]; (iii) Bandura's Social Cognitive Theory (SCT)[30]; and (iv) the *National Health and Medical Research Council Clinical Practice Guidelines for the Management of Obesity in Adults *[31]. The SHED-IT community trial includes two treatment arms:

#### (i) SHED-IT Resources

Participants randomised to this group were provided with a weight loss resource package, which included:

- a 25-minute SHED-IT DVD presentation on weight loss for men;

- the SHED-IT *Weight Loss Handbook for Blokes (NB: 'Blokes' is Australian vernacular meaning men)*;

- the SHED-IT *Weight Loss Support Book for Blokes;*

- a pedometer, tape measure for waist circumference measurement and a kilojoule (kJ) counter book.

The SHED-IT resources have been tailored for men, based on our pilot process evaluation [26] and include examples and scenarios that men can relate to and commonly experience. Both the DVD and *SHED-IT Weight Loss Handbook for Blokes *include basic weight loss information (energy balance, calculating total energy expenditure and tracking energy intake) and outline nine key weight loss messages tailored for men. Through the handbook, men are instructed: to measure and record their weight (in kg) and their waist circumference (in cm) once each week; to complete daily eating and exercise diaries for four days each week (including 2 weekdays and 2 weekend days); to record their step counts for four days each week (including 2 weekdays and 2 weekend days); and to identify and record sources of social support; and personal weight, physical activity and eating goals each month for 3 months. The *SHED-IT Weight Loss Support Book for Blokes *contains instructions for calculating both resting metabolic rate and total kJ expenditure; how to plot weight loss, waist circumference loss and weekly average step charts for recording and monitoring progress; space for recording body weight, physical activity and eating goals; space for recording social support strategies; and a daily food and exercise diary to complete.

#### (ii) SHED-IT Online

In addition to receiving all of the materials from the SHED-IT *Resources *intervention, participants randomised to the SHED-IT *Online *group have access to the freely available commercial *Calorie King*™ (Australia) website (http://www.calorieking.com.au) and a website user guide. *Calorie King*™ is an online, behaviour therapy health website that provides tools and information to help individuals improve their diet and physical activity levels. The *SHED-IT Weight Loss Handbook for Blokes *and the *SHED-IT Weight Loss Support Book for Blokes *were modified slightly with instructions to use the online 'Healthy Lifestyle Diary' for four days each week (including 2 weekdays and 2 weekend days) in place of the paper-based food and exercise diary. Participants were asked to 'weigh-in' on the website at least once a week. As in the SHED-IT *Resources *intervention, men were advised to use their *Weight Loss Support Book for Blokes *to document all other key self-monitoring behaviours, social support strategies and individual goals.

Over the course of the three months, each participant will be emailed seven individualised feedback sheets. Diaries will be reviewed weekly in the 1^st ^month, fortnightly in the 2^nd ^month and once in the 3^rd ^month. This feedback will be provided by research assistants using a standardised set of feedback sheets and will target strategies to address weight loss, reduce energy intake and increase energy expenditure. The feedback is designed to provide general encouragement and specific strategies to address those aspects of the diary entries that are furthest from ideal and/or require the greatest degree of improvement. Participants will be able to record and self-monitor their weight change, energy intake and daily exercise online. These activities are recognised as cornerstones of behavioural treatment [32]. Participants who have not checked-in for 2 weeks will be reminded via email and SMS. Participants are also able to email any questions through to the study email address.

#### (iii)Wait-list control

Men randomised to the control group received no intervention and will be required to attend the assessments at baseline, 3- and 6-month follow up. At the 6-month assessments, these men will then be re-randomised to one of the weight loss groups (*Resources *or *Online*).

### Theoretical Framework of the SHED-IT programs

Achieving and maintaining weight loss requires behaviour change. Interventions that are theoretically-based and evaluated can assist in improving our understanding of the potential mechanisms through which the intervention is working. Bandura's Social Cognitive Theory (SCT) posits that behaviour change is influenced by environmental factors, personal factors, and attributes of the behaviour itself [30]. This interaction is referred to as 'reciprocal determinism', as each factor may affect or be affected by the others. SCT is used as the theory of behaviour change in these interventions as it emphasises changing an individual's cognitions to improve adherence to behaviours that are optional. That is, if men are to change their eating and physical activity behaviours, they must value the outcome (weight loss) of the behaviour, believe they can produce the desired outcome, and believe the outcome will result from successfully completing the behaviour. Our programs target key mediators such as self-efficacy (e.g., knowledge- and skill-based components), self-management (e.g., goal setting, self monitoring), perceived barriers, and social support (i.e., feedback). Both SHED-IT treatment arms are based on the same theoretical constructs, use the same core components to address weight loss and are provided with the same intervention goals for physical activity, diet and weight loss. Table 2 details the specific SHED-IT program content, intervention strategies and alignment with theoretical constructs using the taxonomy of behaviour change strategies identified by Abraham and Michie [33].

**Table 2:** Social Cognitive Theory construct mapping for the SHED-IT interventions

Intervention component	Group allocation	SCT construct	Behaviour change techniques
			* Provide information about behaviour-health link
		* Observational learning	* Use of identifiable role model to model positive behaviours
WL DVD	Resources/Online	* Outcome expectations	* Verbal persuasion from credible information source
		* Behavioural capability	* Prompt self-monitoring of behaviours
		* Self-efficacy	* Prompt specific goal setting
			* Information on consequences

		* Behavioural capability	* Provide information about behaviour-health link
		* Outcome expectations	* Facilitate mastery by encouraging gradual behaviour change
WL Handbook	Resources/Online	* Perceived barriers	* Prompt self-monitoring of behaviours
		* Goal setting & intention	* Prompt specific goal setting (implementation intention)
		* Self-efficacy	* Barrier identification

WL Support Book	Resources/Online	* Self-monitoring * Goal setting * Goal setting * Social support * Intentions * Self-efficacy	* Prompt specific goal setting * Prompt self-monitoring and recording of behaviours (weight chart, waist chart, step count chart, healthy lifestyle diary) * Identification of social support strategies & encourage WL strategies that involve the support of others * Identification of social support strategies & encourage WL strategies that involve the support of others

Self-monitoring items *(kJ counter book, pedometer, tape measure*)		* Self-monitoring	* Facilitate self-monitoring of behaviours
	Resources/Online	* Goal setting	* Prompt specific goal setting (implementation intention)

		* Self-monitoring	* Prompt self-monitoring of behaviour
Access to study website & website user guide	Online only	* Behavioural capability	* Prompt goal setting
		* Goal setting	* Increase knowledge and skills relating to key WL behaviours
		* Self-efficacy	* Detailed instruction of essential website features

		* Social support	* Provide information about behaviour-health link
		* Behavioural capability	* Provide social support and general encouragement
7 individualised feedback sheets	Online only	* Outcome expectations	* Prompt self-monitoring
		* Self-efficacy	* Prompt review of goals & social support strategies

Abbreviations: WL = weight loss; SCT = Social Cognitive Theory; kJ = kilojoule

### Outcomes

Outcome measures were obtained from all participants at baseline (September, 2010) and will be taken at 3 months (December, 2010), and 6 months (March, 2010) after the start of treatment. The primary endpoint will be based on the 6-month follow up measurement. All measurements will be taken in the Human Performance Laboratory at the University of Newcastle (Australia) using the same instruments at each time point. Trained research assistants will adhere to standardised procedures for all data collection and data will be collected in the same order for each time point measurement. Participants were blind to group allocation at the baseline assessment. Assessors will be blinded to treatment allocation at all time points. Participants allocated to the *Control *group will be asked to return to the University for one further assessment, three months after beginning their weight loss programs (9 months after the beginning of the study).

### Demographic characteristics

Background details and sociodemographic variables were collected by questionnaire including age, marital status, occupation, gross annual family income, educational level, ethnic origin, language spoke at home, socioeconomic status (SES) and postcode. SES was based on postal code of residence using the Index of Relative Socioeconomic Advantage and Disadvantage from the Australian Bureau of Statistics census-based Socio-Economic Indexes for Areas (SEIFA)[34].

### Weight

The primary outcome measure is body weight (kg). Weight was measured in light clothing, without shoes on a digital scale to 0.01 kg (CH-150 kp, A&D Mercury Pty Ltd, Australia). Weight was measured twice, with accepted values within 0.1 kg. A third measure was taken if measurements were outside the acceptable range. The average of the two acceptable measures will be reported.

A range of secondary outcome measures were assessed including:

### BMI

BMI was calculated using the standard equation (weight [kg]/height[m]^2^). Height was measured to 0.1 cm using the stretch stature method on 0 a stadiometer (Veeder-Root (VR) High Speed Counter) (Harpenden/Holtain, Mentone Education Centre, Morrabin, Victoria). Height was measured twice, with accepted values within 0.3 cm. A third measure was taken if measurements were outside the acceptable range. The average of the two acceptable measures will be reported.

### Waist circumference

Waist circumference was measured at two points: (i) level with the umbilicus, and (ii) at the largest circumference between the lower costal border and the umbilicus. Two measures were taken at each site, with accepted values within 0.5 cm. Further measures were taken if measurements were outside the acceptable range. The average of the two acceptable measures will be reported. To ensure follow up measurements were taken from the same location, the distances between the sternal notch and both waist circumference points were recorded. Each measurement was recorded with a non-extensible steel tape (KDSF10-02, KDS Corporation, Osaka, Japan). This measure will be taken at each time point by one of two assessors with Level 1 Anthropometry qualifications to improve reliability.

### Blood Pressure and Resting Heart Rate

Blood pressure and resting heart rate were measured using NISSEI/DS-105E digital electronic blood pressure monitors (Nihon Seimitsu Sokki Co. Ltd., Gunma, Japan) under standardised procedures. Participants were seated for five minutes before the first blood pressure measurement and a rest period of two minutes between measures was used. Blood pressure was measured three times. Further measurements were taken if the blood pressure or resting heart rate values fell outside of the acceptable ranges i.e. Systolic within 10 mmHg, diastolic within 10 mmHg (preferably 5 mmHg) and resting heart rate within 5 bpm. The mean of the two closest systolic pressures and the diastolic pressure paired to them will be reported. The mean of the two lowest resting pulse pressures will be used.

### Body composition

Bioimpedance was used for the assessment of body composition, including fat mass, fat free mass and total body water. Body composition was assessed by the InBody720 (Biospace Co., Ltd, Seoul, Korea), a multi-frequency bioimpedance device featuring an eight-point tactile electrode system. This device has been shown to be a valid and reliable device for body composition assessment [35,36].

### Physical activity

Physical activity was objectively measured using pedometers (Yamax SW200 pedometers (Yamax Corporation, Kumamoto City, Japan). Participants were sent pedometers in the mail 1-2 weeks prior to the baseline assessment and will be provided with the pedometer at follow-up assessments. Participants were instructed on how to attach the pedometers (at the waist on the right hand side) and asked to remove the pedometers only when sleeping, when the pedometer might get wet (e.g. swimming, showering) or during contact sports. Participants were asked to wear the pedometers for seven consecutive days and keep to their normal routine. At the end of the day participants were instructed to record their steps on a pedometer record sheet and reset their pedometers to zero. Participants were instructed to note down if they did an activity like cycling, swimming, contact sports or another activity that does not involve stepping and include details (type of activity and duration), or if they forgot to wear their pedometer. Participants will be included in all analyses if they have completed at least four weekdays of pedometer monitoring. The average of existing days will be imputed for participants who have included at least four days of data.

### Dietary Intake

Dietary intake was assessed using the Australian Eating Survey (AES). AES is a 120-item semi-quantitative Food Frequency Questionnaire (FFQ), used previously in Australian youth up to 16 years [37] and currently being validated in both adult males and females. Portion sizes for individual food items were generated by the Australian Bureau of Statistics (ABS) [38] and unpublished data from the 1995 Australian National Nutrition Survey; or the "natural" serving size for common items such as a slice of bread. Subjects were asked about frequency of their consumption over the previous six months with frequency options ranging from 'Never' up to '4 or more times per day' but varying depending on the food item. Twenty-one questions related directly to the intake of vegetables and 11 questions related to fruit. Seasonal availability of some fruits will be considered in the nutrient analysis.

Nutrient intakes from the AES will be computed from the most current food composition database of Australian foods available, the Australian AusNut 1999 database (All Foods) Revision 17 and AusFoods (Brands) Revision 5 (Australian Government Publishing Service, Canberra) to generate individual mean daily macro-and micro-nutrient intakes. The AES includes questions about the total number of daily serves of fruit, vegetables, bread, dairy products, eggs, fat spreads, sweetened beverages and snack foods, as well as asking the type of bread, dairy products and fat spreads used. Twelve questions relate to food-related behaviours, including items on frequency of take-away food consumption and eating while watching television.

### Portion size

Portion size was assessed using portion size photographs from the Dietary Questionnaire for Epidemiological Studies Version 2 (DQES v2), FFQ from the Cancer Council Victoria [39]. These photos are used to calculate a single portion size factor (PSF) to indicate whether on average a person eats median size serves (PSF = 1), more than the median (PSF >1), or less than the median (PSF <1) serve sizes for main meals. The DQES was developed specifically for use in Australian adults by the Cancer Council of Victoria as an update of a FFQ used in a cohort of Australian volunteers aged 40-69 years. Both the development of the questionnaire [40] and its validation have been reported previously [41].

### Alcohol Consumption

Alcohol consumption was measured using an adaptation of the Australian Government Department of Veteran Affairs, Alcohol Use Disorders Identification Test (AUDIT) 2009 [42]. This instrument has been shown to be a valid and reliable measurement tool in determining alcohol use disorders and alcohol misuse [43,44].

### Physical activity and nutrition cognitions

Physical activity and nutrition beliefs were assessed using a number of validated instruments: physical activity self efficacy [45], physical activity outcome expectation [46], physical activity social support [47], physical activity intention [48], nutrition self efficacy [49], nutrition outcome expectations [50], nutrition social support [47] and nutrition intention [48].

### Sedentary Behaviours

Sedentary behaviours were assessed using an adaptation of the Sitting Questionnaire, which has been shown to be both a valid and reliable measure of sitting time in various domains [51,52].

### Quality of Life

Quality of Life and general health was assessed using the UK short form 12 (SF-12) questionnaire [53,54].

### Sleepiness

Daytime Sleepiness was assessed using the Epworth sleepiness scale which is a valid measure of general daytime sleepiness [55].

### Sexual Function

Sexual Function was assessed using the International Index of Erectile Function-5 (IIEF-5) questionnaire which has been shown to be a valid measure of erectile function [56].

### Process measures

Adherence to self-monitoring (total number of daily diet entries, daily exercise entries and weekly weigh-ins) will be calculated from diaries for both treatment arms. In addition to this, men will hand in their SHED-IT support booklet at the 3- and 6-month time points, to be photocopied and posted back. We will also administer a detailed process questionnaire to examine men's perceptions of the SHED-IT program. This will include scales, individual items and open-ended questions that require men to describe the strengths and weaknesses of the program along with their suggestions for improvement. The process evaluation will cover issues such as the study feasibility, opinion of the allocated study group, use and appraisal of components of each intervention and their levels of overall satisfaction. We will also ask how much participants would be willing to pay for the offered intervention. The process evaluation will be administered at the 6-month time point.

### Sample size

The sample size calculation is based on the primary outcome of weight loss at 6 months, which we have assumed will have a standard deviation of 5 kg [24,57]. Thirty six men in each treatment group will give the study 80% power to detect a difference in weight loss between groups of 4 kg at the 1.5% significance level using a two sided test. We have used an alpha of 0.015 to control the Type I error rate for multiple comparisons. A sample size of 150 men was required to allow for an attrition rate of 28%.

### Randomisation

Participants were randomised at an individual level by the trial statistician who will not have any contact with participants during the trial. Allocation was stratified by BMI category calculated at the baseline assessment (overweight, obese I, obese II) and the allocation sequence within strata was generated by a computer-based random number-producing algorithm in block lengths of six. Randomisation codes are stored in a restricted computer folder, which is not accessible by those assessing participants, those involved in group allocating participants or those participating in data entry for the study. Complete separation was achieved between the statistician who generated the randomisation sequence and those who concealed allocation from those involved in implementation of assignments.

### Allocation

Study information for the three different groups was pre-packed into identical black plastic opaque envelopes and consecutively numbered within the three BMI categories and ordered according to the randomisation schedule. The packing and sequencing of these envelopes was completed by a research assistant who was not involved in enrolment, assessment or allocation of participants. Study participants completed all baseline assessments before proceeding to a separate room to meet with a research assistant who was not involved with the baseline assessments. The allocation sequence was concealed during this process. Participants' BMI category was calculated from the baseline measurements and the participant was allocated the next available number in that BMI category before being provided with their information pack. At this point the envelope was opened by the research assistant and details of the particular information pack were provided to the participant using a standardised protocol.

### Data management, quality assurance and exclusion of bias

Randomisation was undertaken by the trial statistician and measures will be taken by trained staff at all times points. In order to ensure accurate and consistent measurements, the study weight scale was professionally calibrated and the height scale checked and recalibrated daily before measurements commenced. All assessments were completed by staff blinded to treatment allocation. When men are contacted (via phone and email) to book in for follow-up assessments they will be asked not to inform data collection personnel of their group allocation. Data will be entered by research assistants blind to group allocation and a program of plausibility checks will be used to identify unrealistic values. The primary outcome measure (weight) will be double entered to ensure accuracy and a random 20% sample of all other measures will also be double entered.

### Statistical methods

Analyses will be performed using Stata Version 11 or later. All variables will be checked for plausibility and missing values. Data will be presented as mean (sd) for continuous variables and counts (percentages) for categorical variables. Differences between groups at randomisation and characteristics of completers versus dropouts will be tested using independent *t *tests for continuous variables and chi-squared (χ^2^) tests for categorical variables. The significance level for the comparison of baseline characteristics will be set at 0.05.

A series of Generalised Linear Mixed Models (GLMMs) with a random intercept for individual will be used to test for differences between treatment groups in the mean level of weight after treatment. Separate models will be fit for each of the pair-wise comparisons (*Resources *vs. *Control*; *Online *vs. *Control*; and *Resources *vs. *Online*). The independent variables in the model will include a variable for treatment group, time (treated as categorical with levels baseline, 3-months and 6-months) and the group-by-time interaction. The model will also include a term for the stratifying variable of BMI group at baseline. The coefficient and p-value for the group-by-6 month interaction term will be used to determine the efficacy of the interventions. Similar models will be used to examine differences in change in other outcome measures.

Using the same approach, additional exploratory models will be used to examine subgroups of the study population. These models will contain the variable that identifies the subgroup (such as SES) and of interest will be the 3 way interaction of treatment group by time by SES. We will also examine a range of secondary outcomes to support the primary outcome (e.g. reduction in waist circumference, increase in physical activity, reduction in kilojoule intake). Additional exploratory models will be fitted to examine if the men who have the greatest reduction in weight are also those who have the greatest improvements in the secondary outcomes.

Statistical significance of the primary efficacy analysis (3 pair-wise comparisons) will be based on Hochberg's multiple testing procedure with the family wise error rate held at 5%. All secondary hypothesis tests will be performed using a 2-sided 5% significance level. In addition, linear regression and GLMMs will be used to describe relationships among the various dependent and independent variables.

A per protocol analysis will also be conducted and include men who complied with treatment from the *Online *and *Resources *component. Men who complied well with the assigned treatment, defined as completion of requested daily eating and exercise diaries (n >40) over the 3-month period and weekly check-ins (n >10). Results of the per-protocol group will be compared with non-compliers in each group i.e. those who did not meet the above adherence recommendations.

## Discussion

The aim of this study is to evaluate the efficacy of two 'low dose' weight loss programs developed specifically for men that could be widely and inexpensively implemented throughout Australia. We will determine whether these innovative approaches to obesity treatment will result in greater initial weight loss and improvements in cardiovascular risk factors compared to a Control group in a community sample of overweight men. There is an urgent need to develop and evaluate novel approaches to weight loss that attract and engage large numbers of men. We will also determine whether web-based support is more effective than resources alone. This study is designed to address the gap in service provision of community-based programs for overweight and obese men. There is limited evidence to guide the design of effective obesity treatment programs for overweight men that would be sustainable in most health care settings that do not require multiple visits to treatment centres.

Our trial targets a national health priority in Australia and focuses on a high-risk under studied population. To successfully combat the obesity epidemic, clinicians and health care systems require feasible, effective and evidence-based treatment options that can be provided to large numbers of men. This randomised controlled trial will test alternative, evidence-based and theoretically driven, easily disseminated strategies specifically for weight loss in men. The interventions are all designed so they could serve as prototypes for rapid translation of research findings into widely available practical applications and widespread implementation in both the public health and medical care sectors. If successful, this project will reduce the negative health, economic and social consequences of obesity through clinically meaningful risk reduction in large numbers of overweight men.

## Competing interests

The authors declare that they have no competing interests.

## Authors' contributions

The study chief investigators PJM, RC, CEC, PM, RCP and Associate Investigator JMW were responsible for identifying the research question, design of the study, obtaining ethics approval, the acquisition of funding and overseeing study implementation. Associate Investigator TB and research assistants NB, MDY, EJA and KLS have contributed to development of intervention materials, recruiting participants and/or study implementation. All authors were responsible for the drafting of this manuscript and have read and approved the final version.

## Pre-publication history

The pre-publication history for this paper can be accessed here:

http://www.biomedcentral.com/1471-2458/10/701/prepub
